# Association between opioid infusion use and duration of mechanical ventilation and related outcomes in critically ill adults: a systematic review and meta-analysis

**DOI:** 10.1016/j.aicoj.2026.100089

**Published:** 2026-05-19

**Authors:** Jia Pei Ong, John W. Devlin, David Culliford, Rebecca Cusack, Michael Grocott, Kinda Ibrahim, Cathrine A. McKenzie

**Affiliations:** a National Institute of Health and Social Care Research (NIHR), Biomedical Research Centre, Southampton, Perioperative and Critical Care Theme, Faculty of Medicine, University of Southampton, United Kingdom; b NIHR Wessex Applied Research Collaborative, University of Southampton, Southampton, United Kingdom; c Pharmacy and Critical Care, University Hospital, Southampton NHS Foundation Trust, Southampton, United Kingdom; d School of Pharmacy and Pharmaceutical Sciences, Bouve College of Health Sciences, Northeastern University, Boston, MA, United States of America; e Division of Pulmonary and Critical Care Medicine, Mass General Brigham, Boston, MA, United States of America

**Keywords:** Opioid, Critical care, Mechanical ventilation, Pain, Delirium, Sedation, Systematic review

## Abstract

**Background:**

We performed a systematic review with meta-analysis to examine the relationship between sedation including a continuous IV opioid infusion and duration of mechanical ventilation (MV) and relevant secondary outcomes in MV critically ill adults.

**Methods:**

We searched MEDLINE, EMBASE, and Cochrane CENTRAL database up to March 2025. We included randomized controlled trials in MV critically ill adults comparing use of sedation including a continuous IV opioid versus sedation without. We assessed MV duration, pain, delirium and coma occurrence, ICU/hospital length of stay (LOS) and 28-day mortality. Study risk of bias was evaluated (PROSPERO: CRD42024498555). We pooled data using a Restricted Maximum Likelihood Estimation random-effects model and followed PRISMA guidelines.

**Results:**

Eight studies (n = 803 patients) published between 2006 and 2021 were included. Sedation including a continuous IV opioid (vs. sedation without) may increase MV duration (3 studies, 425 patients, mean difference (MD) = 3.63 h, 95% confidence interval (CI) 2.27–4.99, very low certainty), reduce pain (2 studies, VAS score at 24 h MD = −0.44 mm, 95% CI − 0.82 to −0.07, low certainty), reduce delirium (3 studies, odds ratio (OR) = 0.28, 95% CI 0.16 to 0.47, very low certainty) and reduce mortality (3 studies, OR = 0.41, 95% CI 0.21 to 0.80, very low certainty). Sedation including a continuous IV opioid does not increase coma or reduce ICU/ hospital LOS. Risk of bias was critical for most studies.

**Conclusion:**

The effect of sedation including a continuous IV opioid on MV duration, pain delirium, coma, mortality and LOS remain uncertain. The role for continuous IV opioids as a part of ICU sedation regimens requires additional evaluation.

## Background

Optimising mechanical ventilation (MV) and ensuring patient comfort are core goals of intensive care unit (ICU) care. Intravenous (IV) opioids, administered as continuous infusions, have served as a foundation for ICU sedation regimens for decades [1,2]. A recent 29-country point prevalence study reported that 87% of ICU adults receive scheduled opioid therapy [3]. A national United Kingdom daily point prevalence study revealed nearly all MV patients receive an opioid (94%) primarily by a continuous infusion [4]. Most of the patients enrolled in the SPICE III (79%) [5], MENDS 2 (69%) [6] A2B (97%) [7] sedation trials were receiving continuous IV opioid infusions at study randomisation.

Important questions about the efficacy and safety of continuous IV opioid therapy in the ICU continue to be raised [8,9]. Patient response is variable; the relationship between the opioid dose and both pain reduction and sedation remain poorly established [10]. Opioid tolerance may occur rapidly, and opioids may exacerbate ventilator dysynchrony rather than help treat it [8]. In the ICU, adverse effects related to opioids are common and often serious. Opioid-associated reductions in gut function may increase abdominal pressure and reduce enteral feeding capacity [8]. Opioids substantially increase the risk for next-day delirium in a dose-dependent fashion, independent of their effect on pain [[11], [12], [13]]. Biologic dependence, occurring as soon as 3 days of therapy, is linked with hyperalgesia, chronic pain syndromes, and may result in long-term use [14,15]. Administration of higher opioid doses during MV are associated with greater persistent opioid use after hospital discharge [9,16]. Long-term opioid exposure in the outpatient setting is an independent risk factor for consequent opioid dependence [17].

Use of continuous IV opioid infusions for the treatment of pain and agitation in the absence of continuous non-opioid sedatives (i.e., analgosedation) in mechanically ventilated adults has been evaluated in multiple randomized controlled trials (RCTs) [[18], [19], [20], [21], [22], [23], [24], [25], [26], [27], [28]] and is guideline-recommended [29]. However, most of these analgosedation RCTs compared continuous IV remifentanil infusions to a different opioid infusion [[18], [19], [20], [21], [22], [23], [24], [25], [26]]. Administering continuous IV opioid infusions to *both* the intervention and control groups, precludes the ability to evaluate the independent effect of a continuous IV opioid infusion on relevant ICU patient outcomes. We were unable to identify a published systematic review (SR) of ICU studies in MV adults that has evaluated the safety and efficacy of a sedation regimen *including* continuously infused IV opioids compared to a sedative regimen *without* continuously-infused IV opioids. Our SR with meta-analysis (MA) therefore examined the relationship between sedation regimens with continuous IV opioids versus sedation regimens without continuous IV opioids on relevant patient outcomes in critically ill adults.

## Methods

### Protocol registration

The study was registered with PROSPERO, CRD4202449855. The MA was performed in line with recommendations from the Cochrane Collaboration and the Preferred Reporting Items for Systematic reviews and Meta-Analyses (PRISMA) Statement [30]. (PRISMA checklist provided in Supplementary Table S1). Institutional review committee approval was not required given that this was a SR and MA of published data.

### Data sources and search strategies

Databases [MEDLINE (OVID), Embase, and the Cochrane Central Register of Controlled Trials] were searched without language or country limitations for randomized controlled trials (RCTs) and quasi RCTs using the search terms, developed in collaboration with a medical librarian, outlined in Supplementary Tables S2-S7, from inception to March 2025. An updated search was conducted in October 2025. We also searched for unpublished studies using the World Health Organization International Clinical Trials Registry and ClinicalTrials.gov database, conference abstracts and proceedings using Web of Science Conference Proceedings Citation Index, and dissertations and theses using Proquest from inception up to March 2025.

### Eligibility criteria

We included RCTs or quasi-controlled trials that examined adults (≥16 years old) admitted to an ICU requiring invasive MV managed with sedation regimen including a continuous IV opioid infusion and compared with a control group managed with a sedation regimen that did not include a continuous IV opioid infusion (but could include intermittent or as needed opioids), and reported on one or more of the following outcomes: duration of MV, pain scores, delirium and coma occurrence, ICU and/or hospital length of stay, short-term mortality. We excluded cohort-control studies, case series and observational studies given their greater potential ROB compared to RCTs.

### Study selection

Two investigators (JO, CM) independently screened the titles and abstracts from the search strategy in Rayyan AI (Rayyan Web and Mobile App for Systematic Review, Cambridge, MA, USA) for potentially relevant studies. The full texts of potentially eligible articles were then assessed for eligibility by the same two investigators. Discrepancies between the two reviewers and discussed collegially with two other investigators (JD, KI) and final decisions regarding inclusion were made by consensus. We also manually examined the references of full-text articles to identify additional potentially relevant trials. Forward citation searching for each included study was conducted using Web of Science. Google Scholar was used for studies not located in the Web of Science. Full text screening of potentially eligible studies identified during title and abstract screening was conducted and their references were examined to identify eligible studies.

### Exposures

Continuously-infused IV opioids refer to the continuous administration of opioids through via a venous catheter. The opioids included in the review (i.e., alfentanil, fentanyl, hydromorphone, morphine, oxycodone, remifentanil, and sufentanil) are the most frequently infused IV opioids in the ICU [3]. Sedatives refer to the IV administration of clonidine, dexmedetomidine, fospropofol, lorazepam, midazolam, propofol or the inhaled use of desflurane, isoflurane, or sevoflurane.

### Outcomes

The outcomes evaluated were chosen based on their inclusion in a recent ICU MV core outcome set [31] and their importance in evaluating the efficacy and safety of continuous IV opioids during MV [9]. The primary outcome, duration of MV, was defined as the time from administration of the continuous opioid following randomisation to the first successful extubation. Pain was categorized based on pain score results [i.e. Behavioural Pain Score (BPS), Critical Care Pain Observation Tool (CPOT), the Numeric Pain Rating Scale (NRS), or the Visual Analogue Scale (VAS)] as follows: no pain (BPS = 3, CPOT = 0, NRS = 0, or VAS = 0 to 4 mm), mild pain (BPS = 4–6, CPOT = 1–3, NRS = 1–3, or VAS = 5–44 mm), moderate pain (BPS = 7–9, CPOT = 3–6, NRS = 4–6, or VAS = 45–74 mm) and severe pain (BPS = 10–12, CPOT = 6–8, NRS = 7–10, or VAS = 75–100 mm) [[32], [33], [34], [35]]. Changes in pain scores were defined as the average change in a pain score over time. Delirium occurred if a Confusion Assessment Method- ICU (CAM-ICU) was positive or an Intensive Care Delirium Screening Checklist score was ≥4 in the ICU at any time following randomisation [36,37]. Whether delirium was prevalent (i.e. first identified at ICU admission or before randomisation) or incident (after randomisation) was not considered. Delirium duration was considered if the study reported it. Coma was defined as a Richmond Agitation Scale (RASS) score of −4 or −5 [38]. Short-term mortality was defined as death within 28 days after randomization. Detailed definitions for the additional outcomes are shown in Supplementary Table S8.

### Data extraction

Two authors (JO and CM) independently extracted data in duplicate using a pre-piloted data abstraction form in Microsoft Excel (Microsoft, Redmond, WA, USA) (Supplemental Table S9). We contacted the corresponding author of a trial publication with missing data. If a corresponding author failed to respond, data available in the publication was used.

### Risk of bias

The quality of the included studies was assessed independently and in duplicate by two reviewers (JO and CM) using the modified version of the Cochrane Collaborative Risk of Bias (RoB) 2 assessment tool [39]. The potential impact of missing data was accounted for in the RoB assessment. Any discrepancies in RoB rating were collegially resolved through discussion with two other investigators (JD, KI). We rated the overall RoB as the highest risk attributable to any domain.

### Certainty of evidence

We assessed the certainty of evidence for each outcome using the Grading of Recommendations Assessment, Development and Evaluation (GRADE) approach [[40], [41], [42]]. We used the Guideline Development Tool (www.guidepro.org) to build the Summary of Findings table and contextualized results using GRADE narrative statements for the five domains (i.e., risk of bias, inconsistency, indirectness, imprecision, and publication bias): high certainty = no qualifiers, moderate certainty = ‘probably’, low certainty = ‘may’, and very lower certainty = ‘an uncertain effect’ [43]. For dichotomous outcomes, we used null as the threshold for imprecision assessment. For the duration of MV, ICU and hospital length of stay, we used one day as the threshold for the imprecision assessment.

### Statistical analysis

We used descriptive statistics to summarise the characteristics of each trial. We used the Restricted Maximum Likelihood Estimation (REML) random-effects meta-analysis model with an inverse variance method to estimate the mean difference for continuous outcomes and pooled odds ratios (OR) for dichotomous outcomes [44]. The Wald-type method was applied to estimate the 95% confidence interval, if the between-study estimates (τ²) were equal to zero [45]. Consistent with Cochrane guidance [45] and the approach used in a recent ICU sedation SR and MA [46], continuous trial outcomes reported as a median with a measure of dispersion were converted into a mean and a standard deviation for outcomes anticipated to have a normal distribution. For outcome’s anticipated to have a skewed distribution, we used the approach recommended by Wan et al. [47]. If there were two studies included for an outcome, a fixed effect inverse variance model was used.

For studies reporting a small event number (≤5) in a group, we used the Mantel-Haenszel fixed-effect model [44]. We assessed statistical heterogeneity using the chi-square test, the I^2^ statistics and visual inspection of the forest plots. Statistically significant heterogeneity was identified if I^2^ was greater than 50% [44]. Publication bias was assessed by visual inspection of funnel plots and Egger’s test if there were more than 10 trials for any outcome [44]. Analyses were performed using RevMan Web version 9.2.1 (Nordic Cochrane Centre, Copenhagen, Denmark) [48].

### Subgroup and sensitivity analyses

Priori subgroup analyses were planned to investigate potential sources of heterogeneity: risk of bias (high versus low), type of ICU population (surgical versus medical), sedative comparator (midazolam versus propofol or dexmedetomidine), opioid use duration (<48 versus ≥48 h) and average opioid daily dose [<200 mg MME (morphine milligram equivalent) or ≥200 mg MME]. The opioid dose was converted to MME based on established clinical guidance [11]. When these subgroup analyses were not possible to conduct, sensitivity analyses were performed to determine and characterise the relationship between different durations and/or doses of opioids with the primary and secondary outcomes.

Sensitivity analyses were also performed to account for any potential overlapping studies by individually excluding each study. Sensitivity analyses for different confidence interval estimation methods (e.g. Hartung-Knapp Sidik-Jonkman) and rare events outcome analysis (e.g. Peto method) were performed for meta-analyses with a small number of studies, given the lack of clear recommendations regarding the most appropriate analytical approach [33]. With dexmedetomidine potentially resulting in different outcomes than midazolam or propofol [46], we also conducted an additional sensitivity analysis of studies using dexmedetomidine vs. midazolam or propofol as the sedative comparator. For statistically significant subgroup findings, we used the Instrument to assess the Credibility of Effect Modification Analyses (ICEMAN) tool to judge subgroup credibility [49]. The ICEMAN tool was employed by two authors independently and in duplicate to judge subgroup credibility, with disagreements resolved by third-party adjudication.

## Results

### Study screening and selection

**Fig. 1:**
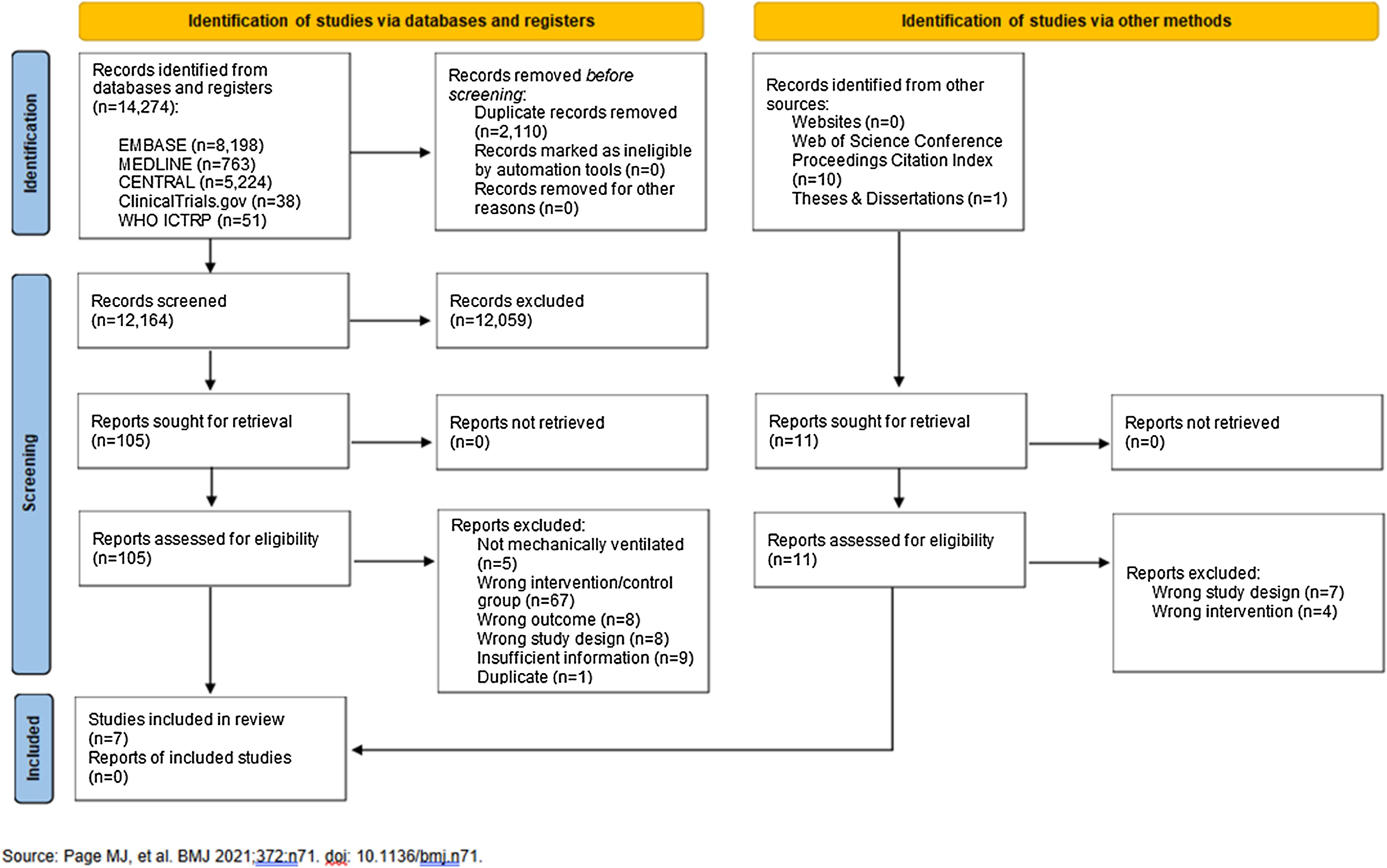
PRISMA flow diagram.

We identified 14,285 articles (14,274 via databases and registers; 11 from other sources) (Fig. 1). We excluded 2,110 articles before screening and 12,059 articles after title and abstract screening. Of the 116 full-text articles/proceedings/theses assessed for eligibility, we included eight studies (n = 834 patients) [[50], [51], [52], [53], [54], [55], [56], [57]]. We subsequently excluded one study after including it, as the opioid exposure was similar between intervention and control groups [57]. Two included studies were conducted in the same centre during a similar period but contained different intervention and control group numbers; the authors did not respond to a clarification request [53,54]. Forward snowballing identified an additional 132 articles of which 23 were considered potentially eligible studies based on title and abstract screening. None of these studies were deemed eligible after full text screening.

### Study characteristics

The seven included studies were all single-centred and published in five countries: USA (n = 2), China (n = 3), Iran (n = 1), and Oman (n = 1) [[50], [51], [52], [53], [54], [55], [56]]. The total number of study participants ranged from 30 to 180 (mean ± SD = 102.4 ± 44.1). The mean participant age was 60.7 ± 3.6 years, and 40.2% were female. The mean baseline Acute Physiology and Chronic Evaluation II (APACHE II) score, reported in three trials [50,53,54], was 21.29 ± 1.6. None of the trials reported a baseline Sequential Organ Failure Assessment (SOFA) or Simplified Acute Physiology Score (SAPS) score. Three studies recruited exclusively post-cardiac surgery patients [51,52,55], two studies recruited surgical patients [53,54] and two studies recruited medical patients [50,56]. Features of the included trials are presented in Table 1.

**Table 1:** Characteristics of included trials.

Study	Country/ sample	Patient characteristics	Inclusion criteria	Intervention group opioids/sedatives	Control group opioids/sedatives	Primary outcome	Funding status
Richman [50]	U.S. n = 30	Mean age: 56.2 yrs Percent female: 50% Mean APACHE II: 23.6	Medical ICU; need for continuous sedation; MV likely >48 h	Fentanyl 0.5 mcg/kg/hr (start dose) titrated to pain/sedation goals + midazolam 0.03 mg/kg/hr (start dose) titrated to sedation goal	Midazolam 0.03 mg/kg/hr (start dose) titrated to sedation goal	Duration of MV	NR
Maddali [51]	Oman n = 180	Mean age: 56.2 yrs Percent female: 28.4% Mean APACHE II: NR	Elective CABG surgery	Fentanyl 0.25−1.5 mcg/kg/hr titrated to pain/sedation goals and propofol 2−5 mg/kg/hr titrated to sedation goal	Propofol 2−5 mg/kg/hr titrated to sedation goal + Diclofenac 75 mg suppository repeated q12 h x once PRN	Duration of MV	NR
Oliver [52]	U.S. n = 145	Mean age: 63.5 yrs Percent female: 36.3% Mean APACHE II: NR	Age 18−90 yrs; elective CABG surgery	Arm 1: Fentanyl 2 mcg/kg/hr (start dose) titrated to pain goal/sedation goal + midazolam IVP PRN agitation Arm 2: Propofol 25−60 mcg/kg/min titrated to sedation goal + fentanyl infusion 0.5 mcg/kg/hr (no titration)	Propofol 25−65 mcg/kg/min titrated to sedation goal and morphine IVP PRN pain	Duration of MV	NR
Lyu [53]	China n = 140	Mean age: 65.3 yrs Percent female: 47.2% Mean APACHE II: 20.2	ICU admission; MV > 24 h; need for continuous sedation infusion	Remifentanil 1 mcg/kg/hr (no titration) + Midazolam 0.02−0.1 mg/kg/hr titrated to sedation goal	Midazolam 0.02−0.1 mg/kg/hr titrated to sedation goal	Delirium	NR
Liu [54]	China n = 105	Mean age: 64.2 yrs Percent female: 47.6% Mean APACHE II: 20.2	Age 18−85 yrs; surgical ICU admission; MV > 24 h; receiving midazolam infusion	Arm 1: Fentanyl 1 mcg/kg/hr (no titration) + midazolam 0.02−0.1 mg/kg/hr titrated to sedation goal Arm 2: Remifentanil 1 mcg/kg/hr (no titration) + midazolam 0.02−0.1 mg/kg/hr titrated to sedation goal	Midazolam 0.02−0.1 mg/kg/hr titrated to sedation goal + placebo opioid infusion (both arms) titrated to pain goal	Delirium	NR
Anvaripour [[55]]	Iran n = 83	Mean age: 58.2 yrs Percent female: 37.3% Mean APACHE II: NR	Age 40−80 yrs; elective CABG surgery	Morphine 0.015 mg/kg/hr (start dose) titrated via PCA	Dexmedetomidine 0.7 mcg/kg/hr (start dose) titrated via PCA	Pain	NR
Wang [[56]]	China n = 70	Mean age: 61.0 yrs Percent female: 50% Mean APACHE II: NR	Severe pneumonia and respiratory failure; requirement for MV	Sufentanil 1 mcg/kg/hr (start dose) titrated to pain goal; propofol 1 mg/kg/hr (start dose) titrated to sedation goal	Propofol 1 mg/kg/hr (start dose) titrated to sedation goal	Pain	NR
Tanios* [57]	U.S. n = 90	Mean age: 65.2 yrs Percent female: 45% Mean APACHE II: 25.2	Expected to require MV ≥48 h; receiving continuous IV midazolam, propofol and/or fentanyl	Fentanyl 25mcg/hr (start dose) and propofol 5 mcg/kg/min as needed for up to 6 h if RASS >1	Midazolam 1 mg/hr (start dose) titrate to sedation goal + fentanyl 25 mg IVP prn pain	Duration of MV	Not funded

APACHE II, Acute Physiologic and Chronic Health Evaluation; CABG, coronary artery bypass graft; CPOT; Clinical Pain Observation Tool; hr, hour; ICU, intensive care unit; IV, intravenous; IVP, intravenous push; kg, kilogram; mcg, microgram; min, minute; MV, mechanical ventilation; NR, not reported; PRN, as needed; Q12 h, every 12 h; RASS, Richmond Agitation Sedation Scale; U.S., United States; yr, years.

* Tanios [57] was excluded from the analysis due to use of fentanyl in the control group that is comparable to the intervention group.

### Summary of interventions

The interventions studied in the seven studies included an IV opioid infusion alone (n = 1), an IV opioid infusion in combination with midazolam (n = 3), an IV opioid infusion in combination with propofol (n = 2) and an IV opioid infusion in combination with dexmedetomidine and/or propofol (n = 1); continuously infused fentanyl was studied in 4 studies, remifentanil in 2 studies, morphine in 1 study, and sufentanil in 1 study; noting one study had three arms. Complete data regarding IV opioid and sedative use, including dose and duration, across intervention and control arms, are summarized in Supplemental Table S10.

### Risk of bias

Supplemental Table S11 reports the RoB assessments. Among the seven studies, six had a high RoB and one had a low RoB. The six studies with a high RoB lacked blinded outcome(s).

### Duration of mechanical ventilation

**Fig. 2:**
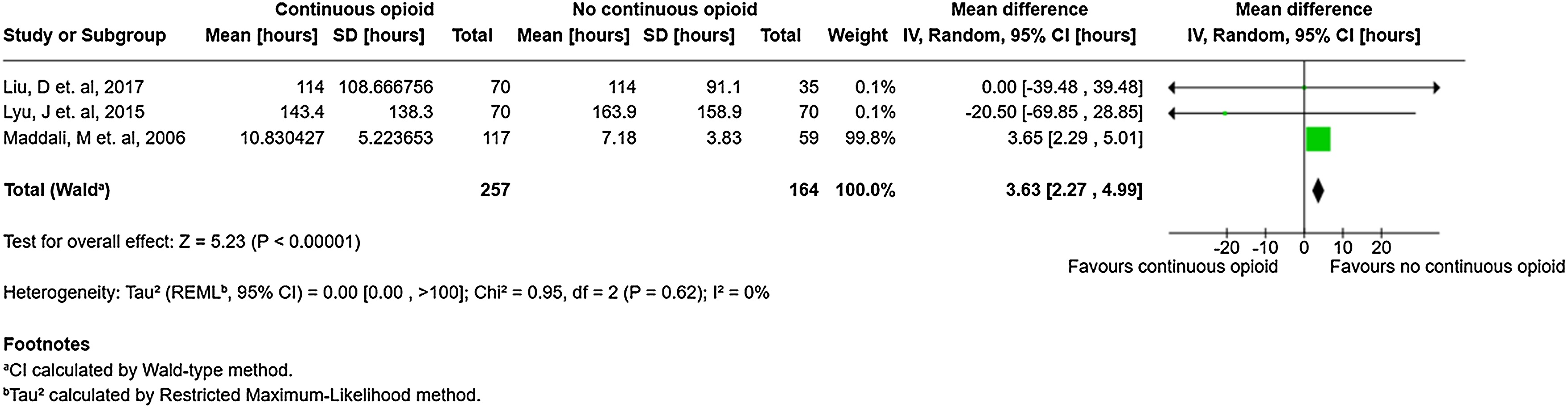
Forest plot examining the relationship between continuous IV opioid (versus no continuous IV opioid) and hours of mechanical ventilation using REML random-effect Wald-type method.

Three studies (n = 421 patients) reported MV duration [51,53,54]. Use of continuous IV opioids was associated with increased MV duration [mean difference (MD) = 3.63 h, 95% CI 2.27–4.99, very low certainty] (Fig. 2). Please also see Supplemental Table S12 (Grade Summary of Findings Table).

### Pain severity

Five studies (n = 547 patients) assessed and reported pain scores [[51], [52], [53], [54],^56^]. Four studies used the VAS [51,52,55,56] and one used CPOT [54]. Four trials reported no statistically significant reduction in pain scores between the intervention and control arms [51,52,54,55]; one trial reported a significant reduction in the pain score in the intervention arm [56]. However, this difference was not associated with a difference in a pain severity category; the average VAS in both arms was in the mild pain category (Supplemental Table S13). Two studies reported VAS score at 24 h after randomisation [52,55]. In the meta-analysis of the two studies, continuous IV opioids were associated with a lower mean VAS score (2 studies, n = 153 patients, MD = −0.44 mm, 95% CI = −0.82 to −0.07, low certainty) (Supplemental Fig. S1).

### Delirium occurrence

**Fig. 3:**
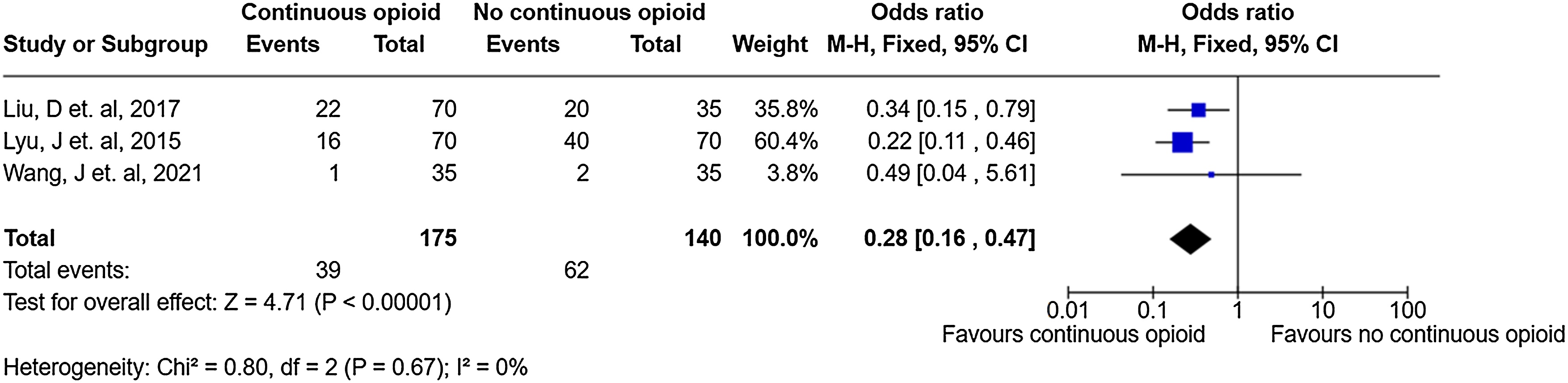
Forest plot examining the relationship between continuous IV opioid (versus non-continuous IV opioid) and delirium occurrence using Mantel–Haenszel fixed effect methods.

Three trials (n = 315 patients) reported delirium occurrence [53,54,56]. Use of continuous IV opioids may reduce delirium occurrence [odds ratio (OR) = 0.28, 95% CI = 0.16 to 0.47, very low certainty] (Fig. 3 and Supplemental Table S12). For two of these three trials (n = 245 patients) where delirium duration was reported [53,54], continuous IV opioid use was not associated with a difference in time spent with delirium (MD = −22.49, 95% CI −49.86 to 4.88) (Supplemental Fig. S2).

### Coma occurrence

Only one trial (n = 30 patients) evaluated the occurrence of coma (not related to an underlying intracranial process) and reported no statistically significant difference in coma between patients receiving fentanyl and midazolam versus midazolam alone (Supplemental Table S12) [50].

### ICU and hospital length of stay

Three studies (n = 358 patients) reported ICU length of stay [[52], [53], [54]]; one study (n = 113) reported hospital length of stay [52]. Continuous IV opioid use was not associated with a difference in either ICU (MD = 0 days, 95% CI −0.03 to 0.04, very low certainty) (Supplemental Fig. S3 and Supplemental Table S12) or hospital length of stay (median = 6 days, range 5–8, p = 0.39) (Supplemental Table S12).

### Short-term mortality

**Fig. 4:**
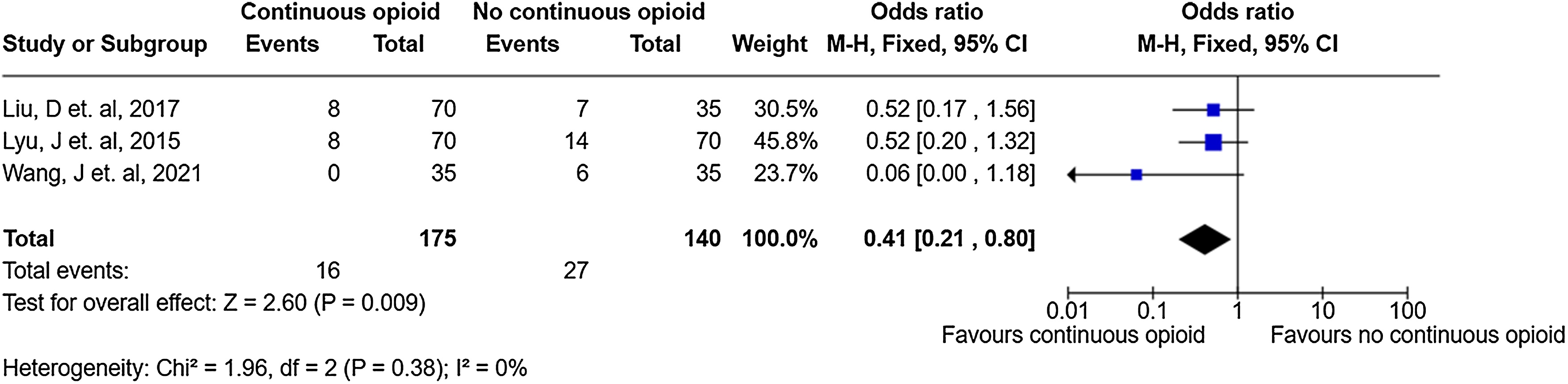
Forest plot examining the relationship between continuous IV opioid (versus non-continuous IV opioid) and short-term mortality using Mantel–Haenszel fixed effect methods.

Three trials (n = 315 patients) reported short-term mortality [53,54,56]. Continuous IV opioid use was associated with a reduction in short-term mortality (OR = 0.41, 95% CI 0.21 to 0.80), very low certainty) (Fig. 4 and Supplemental Table S12).

### Sensitivity analyses and subgroup analyses

For the three studies evaluating MV duration, use of the Hartung-Knapp-Sidik-Jonkman method (in the sensitivity analysis) increased the summary confidence interval but did not change the MD (MD = 3.63 h, 95% CI 1.57–5.69) (Supplemental Fig. S4). Neither the MV duration or delirium occurrence results changed when the Peto (vs. Mantel-Haenszel) method was used to pool the odds ratios for rare events (Supplemental Figures S5 and S6). A statistical difference in duration of MV was not found when studies evaluating only cardiac surgery patients that used a continuous IV opioid ≤48 h or administered an average daily opioid dose ≤200 mg MME were excluded (Supplemental Table S14). Further sensitivity analyses reported no difference between continuous IV opioids and control groups for patient type, dexmedetomidine or propofol use in the comparator group, and duration and dose of IV opioid used (Supplemental Table S14). A sensitivity analysis examining the impact of dexmedetomidine only in the comparator group could not be completed as the study in which dexmedetomdine was used solely as the comparator [56] reported VAS scores 24 h after randomization and only one other study [56] reporting this same outcome. Removal of either of the potentially two overlapping studies [53,54] significantly increased the I^2^ statistic (I^2^ = 47%) for short-term mortality, suggesting moderate heterogeneity between these two studies for mortality may exist (Supplemental Table S15). The high (vs. low) risk of bias analysis could not be completed, given 6/7 studies were rated as having a high risk of bias.

### Publication bias

Publication bias was not assessed using formal statistical methods (i.e. funnel plots or Egger regression), due to an inadequate number of studies evaluating each outcome.

## Discussion

The administration of continuous IV opioids is common in MV adults despite a lack of strong evidence demonstrating their benefit in a setting where important short and long-term safety concerns exist [8]. However, most of the RCTs used to support the current PADIS 2018 recommendation to use an assessment-driven analgesia/analgosedation, protocol-based, stepwise approach, for pain and sedation management in critically ill adults [29] administered a continuous IV opioid infusion to both the intervention and control groups [[18], [19], [20], [21], [22], [23], [24], [25], [26], [27],^57^]. Our SR and MA, by focusing on the comparative efficacy and safety of an ICU sedation approach that includes continuous IV opioid administration (versus one that does not) suggests the effect of this approach on duration of MV, pain, delirium, mortality and length of stay remains uncertain. However, the seven studies we identified were relatively small, conducted at a single-centre, and had a high risk of bias. Three of the studies were published more than a decade ago [[50], [51], [52]], a period when daily spontaneous awakening/breathing trials were not well-established [58] and three of the studies [51,52,55] enrolled cardiac surgery patients, a population with a lower severity of illness and that often transitions through the ICU quickly. Lastly, only two of the trials [50,51] were conducted in higher resource countries (e.g., U.S., Europe) where ICU sedation practices may be different than lower resource countries [59].

Duration of MV was selected as our primary outcome given its importance as a core ICU research outcome [31] and the premise that if opioids improve patient comfort and ventilator tolerance, liberation from MV may be expedited [8]. Instead, use of continuous IV opioid infusions in ICU sedation regimens may increase MV duration. Although the difference we found was small and the high risk of bias across studies makes this result uncertain, our result remains potentially concerning given duration of MV is associated with increased pneumonia, venous thrombosis and healthcare costs [58,60]. While we were not able to detect a difference in opioid dose or duration in our limited sensitivity analyses, an association between continuous IV opioid dose and duration and MV duration may be a result of increased opioid sedation and respiratory depression, each of which may reduce the success of spontaneous awakening and breathing trials.

Maintaining patient comfort in the ICU is a fundamental goal of critical care; IV opioids are recommended as the first-line analgesic strategy in the 2013 PAD guidelines [2]. Among the five studies in our review that assessed and reported pain scores [[51], [52], [53], [54],^56^], four studies did not report a difference in level of pain between the IV opioid infusion and non-IV opioid infusion groups [51,52,54,55]. In the one study that reported lower pain scores with continuous IV opioid use [56], the difference between the groups was small and pain scores in both groups remained within the mild pain category, suggesting limited clinical significance. This result is different from a summary of the five analgosedation RCTs [19,20,26,28,61] used to support the 2018 PADIS guideline recommendation that an assessment-driven, protocolized analgesia/analgosedation-driven approach be used in critically ill adults [29]. Across these studies, pain severity was found to be significantly reduced in the intervention (i.e., IV opioid infusion) arm. However, four of these RCTs administered IV opioid infusions to both the intervention and control arm [19,20,26,28], and in the one RCT where the control group did not receive an IV opioid infusion, daily opioid exposure was similar between the intervention and control groups [61].

The reduction in delirium occurrence with continuous IV opioid infusion use is not consistent with recent evidence demonstrating that ICU opioid exposure is independently associated with increased delirium [11,13]. Although the relationship between ICU pain and delirium remains controversial [12,13], among the three studies reporting delirium [53,54,56], only two reported pain. Further, two of these studies [53,54] compared continuous opioids to continuous midazolam: another well-established risk factor for ICU delirium [62].

Only three studies reported short-term mortality [53,54,56]. Two of these studies evaluated midazolam as the comparator [53,54] and one evaluated dexmedetomidine or propofol as the comparator [56]. The heterogeneity between sedation choice among these three studies and evidence suggesting patient age may influence sedation choice-associated mortality [63], makes conclusions about an association between opioid infusion use and mortality challenging to make.

Choice of opioid has been postulated to influence the pain response [8]. One recent ICU RCT found that analgosedation with continuous fentanyl was associated with an increase in ventilator- free days [64] yet greater delirium [65] compared to continuous morphine. A summary of RCTs comparing remifentanil vs fentanyl ICU analgosedation failed to demonstrate differences in opioid effectiveness and related outcomes [66,67]. A lack of comparative evidence exists for alfentanil and hydromorphone, two of the most widely used opioids in ICUs [3]. Given the variety of IV opioids used in the studies we included in our SR and MA, we were not able to conduct additional subgroup analyses to explore whether choice of opioid influenced the results we report.

Given the methodological limitations and low or very low certainty of the evidence reported in this SR and MA, we can neither endorse nor recommend against the administration of IV opioid infusions as part of a sedation regimen in MV adults. While pain should be routinely assessed and managed in MV adults, our results do not support the routine initiation of continuous IV opioid infusions to treat pain or agitation or improve ventilator tolerance over intermittent or ‘as-needed’ opioid and non-opioid analgesic therapy. Multimodal analgesia approaches may help improve pain control and reduce opioid exposure in patients admitted to the ICU after major surgery or trauma [29].

Our review has several strengths. In most of the large analgosedation RCTs published to date both the intervention and control groups received continuous IV opioid infusions [[18], [19], [20], [21], [22], [23], [24], [25], [26]]. As far as we are aware, our paper is the first SR and MA to rigorously explore the evidence regarding the efficacy and safety of IV opioid infusions as part of sedation regimens in MV adults. We were able to explore multiple different patient-centric outcomes, many of which are recommended by ICU core outcome sets. Our methods were rigorous and followed PRISMA guidelines. We conducted relevant sensitivity and sub-group analysis where possible.

A key limitation of the results we report are the small number of trials we identified and their limited samples thus potentially leading to statistical imprecision and a lack of generalizability to the broader MV ICU population. The effect of publication bias on our results remains unclear given it could not be analysed. The high risk of bias in the studies we included resulted in low to very low certainty evidence for all outcomes evaluated. None of the studies reported duration of MV as ventilator-free days despite ICU mortality being an important competing risk for this outcome in the ICU [68]. Pain, delirium and coma assessments was conducted by bedside clinicians rather than blinded researchers. Further, opioid daily dosing was not reported and therefore not considered. Many of the studies did not fully report key methods and results; authors did not respond to requests for additional data and clarification despite multiple attempts. Indirectness is important concern in our analysis given most studies did not use ‘standard of care’ as the comparator group, which may affect the clinical applicability of our findings.

## Conclusion

Despite conducting a rigorous SR and MA of ICU RCTs evaluating the use of a continuous IV opioid infusion in MV patients as part of a sedation regimen, we are unable to establish whether use of continuous opioid infusions influence the duration of MV or other important secondary clinical outcomes. Large multicentre RCTs that include MV adults who are critically ill and managed at a light sedation goal with either propofol or dexmedetomidine are needed to evaluate the efficacy and safety of the adding continuous opioid infusions to sedation regimens in MV adults.

## Authors' contributions

JPO, JWD and CAM contributed to study conception and design. Material preparation, data collection and analysis were performed by JPO, JWD, DC, and CAM. DC provided expert statistical input throughout. The first draft of the manuscript was written by JPO, JWD, and CAM. MG gave senior support. KI and RC were third arbiter and gave clinical expertise respectively. All authors reviewed the final manuscript and resubmission.

## Consent for publication

Not applicable.

## Ethics approval and consent to participate

Not applicable.

## Funding

The efforts of JPO was supported by a NIHR predoctoral bridging award and the efforts of CAM was supported by NIHR Senior Clinical Practitioner Research AwardNIHR304615.

## Availability of data and material

Not applicable.

## Declaration of competing interest

None of the authors received support from any organization for the submitted work; have a financial relationship with any organization that might have an interest in the submitted work in the prior three years; or have other relationships or activities that could appear to have influenced the submitted work.
