# Supplementary material for: Association between opioid infusion use and duration of mechanical ventilation and related outcomes in critically ill adults: a systematic review and meta-analysis
Source: Ann Intensive Care. 2026 May 19;16:100089. doi: 10.1016/j.aicoj.2026.100089 (PMC13218110; doi:10.1016/j.aicoj.2026.100089)
Supplement: Supplementary file 1 [file mmc1.docx]

**Supplemental Material**

**Supplemental Table 1. PRISMA 2020 Checklist**

| **Section and Topic** | **Item #** | **Checklist item** | **Location where item is reported** |
| --- | --- | --- | --- |
| **TITLE** | | |  |
| Title | 1 | Identify the report as a systematic review. | 1 |
| **ABSTRACT** | | |  |
| Abstract | 2 | See the PRISMA 2020 for Abstracts checklist. | 2 |
| **INTRODUCTION** | | |  |
| Rationale | 3 | Describe the rationale for the review in the context of existing knowledge. | 3 |
| Objectives | 4 | Provide an explicit statement of the objective(s) or question(s) the review addresses. | 4 |
| **METHODS** | | |  |
| Eligibility criteria | 5 | Specify the inclusion and exclusion criteria for the review and how studies were grouped for the syntheses. | 4-5 |
| Information sources | 6 | Specify all databases, registers, websites, organisations, reference lists and other sources searched or consulted to identify studies. Specify the date when each source was last searched or consulted. | 4 |
| Search strategy | 7 | Present the full search strategies for all databases, registers and websites, including any filters and limits used. | 4 and Suppl Table 2-7 |
| Selection process | 8 | Specify the methods used to decide whether a study met the inclusion criteria of the review, including how many reviewers screened each record and each report retrieved, whether they worked independently, and if applicable, details of automation tools used in the process. | 5-6 |
| Data collection process | 9 | Specify the methods used to collect data from reports, including how many reviewers collected data from each report, whether they worked independently, any processes for obtaining or confirming data from study investigators, and if applicable, details of automation tools used in the process. | 5-6 |
| Data items | 10a | List and define all outcomes for which data were sought. Specify whether all results that were compatible with each outcome domain in each study were sought (e.g. for all measures, time points, analyses), and if not, the methods used to decide which results to collect. | 4-5 |
|  | 10b | List and define all other variables for which data were sought (e.g. participant and intervention characteristics, funding sources). Describe any assumptions made about any missing or unclear information. | Appendix S8 |
| Study risk of bias assessment | 11 | Specify the methods used to assess risk of bias in the included studies, including details of the tool(s) used, how many reviewers assessed each study and whether they worked independently, and if applicable, details of automation tools used in the process. | 6 |
| Effect measures | 12 | Specify for each outcome the effect measure(s) (e.g. risk ratio, mean difference) used in the synthesis or presentation of results. | 5-7 |
| Synthesis methods | 13a | Describe the processes used to decide which studies were eligible for each synthesis (e.g. tabulating the study intervention characteristics and comparing against the planned groups for each synthesis (item #5)). | 6-7 |
|  | 13b | Describe any methods required to prepare the data for presentation or synthesis, such as handling of missing summary statistics, or data conversions. | 6-8 |
|  | 13c | Describe any methods used to tabulate or visually display results of individual studies and syntheses. | 6-8 |
|  | 13d | Describe any methods used to synthesize results and provide a rationale for the choice(s). If meta-analysis was performed, describe the model(s), method(s) to identify the presence and extent of statistical heterogeneity, and software package(s) used. | 6-8 |
|  | 13e | Describe any methods used to explore possible causes of heterogeneity among study results (e.g. subgroup analysis, meta-regression). | 7-8 |
|  | 13f | Describe any sensitivity analyses conducted to assess robustness of the synthesized results. | 7-8 |
| Reporting bias assessment | 14 | Describe any methods used to assess risk of bias due to missing results in a synthesis (arising from reporting biases). | 7-8 |
| Certainty assessment | 15 | Describe any methods used to assess certainty (or confidence) in the body of evidence for an outcome. | 7 |
| **RESULTS** | | |  |
| Study selection | 16a | Describe the results of the search and selection process, from the number of records identified in the search to the number of studies included in the review, ideally using a flow diagram. | 8-9 |
|  | 16b | Cite studies that might appear to meet the inclusion criteria, but which were excluded, and explain why they were excluded. | 9 |
| Study characteristics | 17 | Cite each included study and present its characteristics. | 9-10 |
| Risk of bias in studies | 18 | Present assessments of risk of bias for each included study. | 9 |
| Results of individual studies | 19 | For all outcomes, present, for each study: (a) summary statistics for each group (where appropriate) and (b) an effect estimate and its precision (e.g. confidence/credible interval), ideally using structured tables or plots. | 9-11 |
| Results of syntheses | 20a | For each synthesis, briefly summarise the characteristics and risk of bias among contributing studies. | 9-11 |
|  | 20b | Present results of all statistical syntheses conducted. If meta-analysis was done, present for each the summary estimate and its precision (e.g. confidence/credible interval) and measures of statistical heterogeneity. If comparing groups, describe the direction of the effect. | 9-11 |
|  | 20c | Present results of all investigations of possible causes of heterogeneity among study results. | 9-11 |
|  | 20d | Present results of all sensitivity analyses conducted to assess the robustness of the synthesized results. | 11 |
| Reporting biases | 21 | Present assessments of risk of bias due to missing results (arising from reporting biases) for each synthesis assessed. | 9-11 |
| Certainty of evidence | 22 | Present assessments of certainty (or confidence) in the body of evidence for each outcome assessed. | 9-11 |
| **DISCUSSION** | | |  |
| Discussion | 23a | Provide a general interpretation of the results in the context of other evidence. | 12-15 |
|  | 23b | Discuss any limitations of the evidence included in the review. | 12-15 |
|  | 23c | Discuss any limitations of the review processes used. | 12-15 |
|  | 23d | Discuss implications of the results for practice, policy, and future research. | 12-15 |
| **OTHER INFORMATION** | | |  |
| Registration and protocol | 24a | Provide registration information for the review, including register name and registration number, or state that the review was not registered. | 4 |
|  | 24b | Indicate where the review protocol can be accessed, or state that a protocol was not prepared. | 4 |
|  | 24c | Describe and explain any amendments to information provided at registration or in the protocol. | 4 |
| Support | 25 | Describe sources of financial or non-financial support for the review, and the role of the funders or sponsors in the review. | 17 |
| Competing interests | 26 | Declare any competing interests of review authors. | 17 |
| Availability of data, code and other materials | 27 | Report which of the following are publicly available and where they can be found: template data collection forms; data extracted from included studies; data used for all analyses; analytic code; any other materials used in the review. | Supplements |

*From:*  Page MJ, McKenzie JE, Bossuyt PM, Boutron I, Hoffmann TC, Mulrow CD, et al. The PRISMA 2020 statement: an updated guideline for reporting systematic reviews. BMJ 2021;372:n71. doi: 10.1136/bmj.n71. This work is licensed under CC BY 4.0. To view a copy of this license, visit [https://creativecommons.org/licenses/by/4.0/](https://protect.checkpoint.com/v2/r06/___https://creativecommons.org/licenses/by/4.0/___.ZXV3Mjp1bml2ZXJzaXR5aG9zcGl0YWxzb3V0aGFtcHRvbjpjOm86N2Q0MzgyYjc5NzdkMjUwNGU2N2I0ZDg4ODUzYmJmNWE6NzoxODlkOjEwNzEzYWQwOWE4ZTg1OGE5NGVmZjU1NjY2NjcwOTA0MmEwNTZhNDIwODBiMTM3OGI2YWNlZDBlNDJjMjI5ZDI6cDpUOk4)

**Supplemental Table 2. MEDLINE Search Strategy**

1 morphine.mp. [mp=title, book title, abstract, original title, name of substance word, subject heading word, floating sub-heading word, keyword heading word, organism supplementary concept word, protocol supplementary concept word, rare disease supplementary concept word, unique identifier, synonyms, population supplementary concept word, anatomy supplementary concept word] (67471)

2 exp Morphine/ (41193)

3 1 or 2 (67471)

4 fentanyl.mp. or exp Fentanyl/ (30370)

5 sufentanil.mp. or exp Sufentanil/ (3973)

6 exp Alfentanil/ (1741)

7 alfentanil.mp. [mp=title, book title, abstract, original title, name of substance word, subject heading word, floating sub-heading word, keyword heading word, organism supplementary concept word, protocol supplementary concept word, rare disease supplementary concept word, unique identifier, synonyms, population supplementary concept word, anatomy supplementary concept word] (2550)

8 6 or 7 (2550)

9 remifentanil.mp. [mp=title, book title, abstract, original title, name of substance word, subject heading word, floating sub-heading word, keyword heading word, organism supplementary concept word, protocol supplementary concept word, rare disease supplementary concept word, unique identifier, synonyms, population supplementary concept word, anatomy supplementary concept word] (6750)

10 exp Remifentanil/ (4052)

11 9 or 10 (6750)

12 hydromorphone.mp. or exp Hydromorphone/ (2712)

13 methadone.mp. or Methadone/ (19841)

14 opioid$.mp. or exp Analgesics, Opioid/ (218914)

15 opiate$.mp. or exp Opiate Alkaloids/ (115702)

16 14 or 15 (242590)

17 oxycodone.mp. or exp Oxycodone/ (5700)

18 3 or 4 or 5 or 8 or 11 or 12 or 13 or 16 or 17 (263734)

19 ventilat*.mp. [mp=title, book title, abstract, original title, name of substance word, subject heading word, floating sub-heading word, keyword heading word, organism supplementary concept word, protocol supplementary concept word, rare disease supplementary concept word, unique identifier, synonyms, population supplementary concept word, anatomy supplementary concept word] (243331)

20 ((mechan* or artific*) adj3 (respirat* or ventilat*)).mp. [mp=title, book title, abstract, original title, name of substance word, subject heading word, floating sub-heading word, keyword heading word, organism supplementary concept word, protocol supplementary concept word, rare disease supplementary concept word, unique identifier, synonyms, population supplementary concept word, anatomy supplementary concept word] (139307)

21 exp Respiration, Artificial/ (93322)

22 exp Ventilators, Mechanical/ (10458)

23 exp Continuous Positive Airway Pressure/ or cpap.mp. (15922)

24 ncpap.mp. or Positive-Pressure Respiration/ (19734)

25 APRV.mp. (234)

26 airway pressure release ventilat*.mp. [mp=title, book title, abstract, original title, name of substance word, subject heading word, floating sub-heading word, keyword heading word, organism supplementary concept word, protocol supplementary concept word, rare disease supplementary concept word, unique identifier, synonyms, population supplementary concept word, anatomy supplementary concept word] (361)

27 respirat*.mp. [mp=title, book title, abstract, original title, name of substance word, subject heading word, floating sub-heading word, keyword heading word, organism supplementary concept word, protocol supplementary concept word, rare disease supplementary concept word, unique identifier, synonyms, population supplementary concept word, anatomy supplementary concept word] (908141)

28 IPPV.mp. or exp Intermittent Positive-Pressure Ventilation/ (2885)

29 interactive ventilator support.mp. (1)

30 neurally adjusted ventilat*.mp. [mp=title, book title, abstract, original title, name of substance word, subject heading word, floating sub-heading word, keyword heading word, organism supplementary concept word, protocol supplementary concept word, rare disease supplementary concept word, unique identifier, synonyms, population supplementary concept word, anatomy supplementary concept word] (453)

31 19 or 20 or 21 or 22 or 23 or 24 or 25 or 26 or 27 or 28 or 29 or 30 (1026043)

32 ICU.mp. or exp Critical Illness/ (129287)

33 MICU.mp. (1040)

34 SICU.mp. (988)

35 exp Coronary Care Units/ or exp Intensive Care Units/ or CICU.mp. (115860)

36 ((intensive* or critical*) adj3 (care* or treat*)).mp. [mp=title, book title, abstract, original title, name of substance word, subject heading word, floating sub-heading word, keyword heading word, organism supplementary concept word, protocol supplementary concept word, rare disease supplementary concept word, unique identifier, synonyms, population supplementary concept word, anatomy supplementary concept word] (342936)

37 exp Critical Care/ (69624)

38 intensive care unit.mp. (150602)

39 intensive care.mp. (256647)

40 critical care.mp. (97213)

41 32 or 33 or 34 or 35 or 36 or 37 or 38 or 39 or 40 (386999)

42 18 and 31 and 41 (2435)

43 ((control* or clinical*) adj3 trial).mp. [mp=title, book title, abstract, original title, name of substance word, subject heading word, floating sub-heading word, keyword heading word, organism supplementary concept word, protocol supplementary concept word, rare disease supplementary concept word, unique identifier, synonyms, population supplementary concept word, anatomy supplementary concept word] (1244307)

44 exp Randomized Controlled Trial/ or exp Clinical Trial/ or trial.mp. (1515946)

45 clinical trial.mp. (838803)

46 exp Random Allocation/ or random*.mp. (1863253)

47 therap*.mp. (7832535)

48 drug.mp. (6732849)

49 43 or 44 or 45 or 46 or 47 or 48 (12145514)

50 42 and 49 (1859)

51 (intravenous or (Administration, Intravenous or Administrations, Intravenous or Drip, Intravenous or INTRAVENOUS INJECT or Infusion, Intravenous or Infusions, Intravenous or Injection, Intravenous or Injections, Intravenous or Intravenous Administration or Intravenous Administrations or Intravenous Drug Delivery System or Intravenous Drug Delivery Systems or Intravenous Infusion or Intravenous Infusions or Intravenous Injection or Intravenous Injections)).mp. [mp=title, book title, abstract, original title, name of substance word, subject heading word, floating sub-heading word, keyword heading word, organism supplementary concept word, protocol supplementary concept word, rare disease supplementary concept word, unique identifier, synonyms, population supplementary concept word, anatomy supplementary concept word] (452511)

52 (Infusion, Parenteral or Infusions, Parenteral or Parenteral Infusion or Parenteral Infusions).mp. (26766)

53 intravenous.mp. or exp Injections, Intravenous/ or exp Administration, Intravenous/ or exp Infusions, Intravenous/ (452511)

54 exp Infusions, Parenteral/ or parenteral.mp. (160389)

55 (Drug Infusion System or Drug Infusion Systems or Infusion System, Drug or Infusion Systems, Drug or Infusion, Drip or Pump, Infusion).mp. (372)

56 infusion.mp. (259552)

57 51 or 52 or 53 or 54 or 55 or 56 (702217)

58 50 and 57 (763)

**Supplemental Table 3. EMBASE Search Strategy**

**1**  morphine.mp. [mp=title, abstract, heading word, drug trade name, original title, device manufacturer, drug manufacturer, device trade name, keyword heading word, floating subheading word, candidate term word] (141901)
**2**  exp Morphine/ (122062)
**3**  1 or 2 (141901)
**4**  fentanyl.mp. or exp Fentanyl/ (93219)
**5**  sufentanil.mp. or exp Sufentanil/ (14576)
**6**  exp Alfentanil/ (7427)
**7**  alfentanil.mp. [mp=title, abstract, heading word, drug trade name, original title, device manufacturer, drug manufacturer, device trade name, keyword heading word, floating subheading word, candidate term word] (7634)
**8**  6 or 7 (7634)
**9**  remifentanil.mp. [mp=title, abstract, heading word, drug trade name, original title, device manufacturer, drug manufacturer, device trade name, keyword heading word, floating subheading word, candidate term word] (21685)
**10**  exp Remifentanil/ (21109)
**11**  9 or 10 (21685)
**12**  hydromorphone.mp. or exp Hydromorphone/ (14736)
**13**  methadone.mp. or Methadone/ (43521)
**14**  opioid$.mp. or exp Analgesics, Opioid/ (490455)
**15**  opiate$.mp. or exp Opiate Alkaloids/ (210971)
**16**  14 or 15 (512785)
**17**  oxycodone.mp. or exp Oxycodone/ (27769)
**18**  3 or 4 or 5 or 8 or 11 or 12 or 13 or 16 or 17 (521682)
**19**  ventilat*.mp. [mp=title, abstract, heading word, drug trade name, original title, device manufacturer, drug manufacturer, device trade name, keyword heading word, floating subheading word, candidate term word] (482584)
**20**  ((mechan* or artific*) adj3 (respirat* or ventilat*)).mp. [mp=title, abstract, heading word, drug trade name, original title, device manufacturer, drug manufacturer, device trade name, keyword heading word, floating subheading word, candidate term word] (251436)
**21**  exp Respiration, Artificial/ (317532)
**22**  exp Ventilators, Mechanical/ (7996)
**23**  exp Continuous Positive Airway Pressure/ or cpap.mp. (32319)
**24**  ncpap.mp. or Positive-Pressure Respiration/ (5774)
**25**  APRV.mp. (531)
**26**  airway pressure release ventilat*.mp. [mp=title, abstract, heading word, drug trade name, original title, device manufacturer, drug manufacturer, device trade name, keyword heading word, floating subheading word, candidate term word] (788)
**27**  respirat*.mp. [mp=title, abstract, heading word, drug trade name, original title, device manufacturer, drug manufacturer, device trade name, keyword heading word, floating subheading word, candidate term word] (1470484)
**28**  IPPV.mp. or exp Intermittent Positive-Pressure Ventilation/ (4635)
**29**  interactive ventilator support.mp. (1)
**30**  neurally adjusted ventilat*.mp. [mp=title, abstract, heading word, drug trade name, original title, device manufacturer, drug manufacturer, device trade name, keyword heading word, floating subheading word, candidate term word] (767)
**31**  19 or 20 or 21 or 22 or 23 or 24 or 25 or 26 or 27 or 28 or 29 or 30 (1742963)
**32**  ICU.mp. or exp Critical Illness/ (221992)
**33**  MICU.mp. (3573)
**34**  SICU.mp. (2110)
**35**  exp Coronary Care Units/ or exp Intensive Care Units/ or CICU.mp. (347064)
**36**  ((intensive* or critical*) adj3 (care* or treat*)).mp. [mp=title, abstract, heading word, drug trade name, original title, device manufacturer, drug manufacturer, device trade name, keyword heading word, floating subheading word, candidate term word] (617886)
**37**  exp Critical Care/ (972071)
**38**  intensive care unit.mp. (381571)
**39**  intensive care.mp. (545754)
**40**  critical care.mp. (84089)
**41**  32 or 33 or 34 or 35 or 36 or 37 or 38 or 39 or 40 (1357213)
**42**  18 and 31 and 41 (27379)
**43**  ((control* or clinical*) adj3 trial).mp. [mp=title, abstract, heading word, drug trade name, original title, device manufacturer, drug manufacturer, device trade name, keyword heading word, floating subheading word, candidate term word] (2487210)
**44**  exp Randomized Controlled Trial/ or exp Clinical Trial/ or trial.mp. (2997450)
**45**  clinical trial.mp. (1906615)
**46**  exp Random Allocation/ or random*.mp. (2482559)
**47**  therap*.mp. (11444839)
**48**  drug.mp. (13894856)
**49**  43 or 44 or 45 or 46 or 47 or 48 (19448862)
**50**  42 and 49 (24068)
**51**  (intravenous or (Administration, Intravenous or Administrations, Intravenous or Drip, Intravenous or INTRAVENOUS INJECT or Infusion, Intravenous or Infusions, Intravenous or Injection, Intravenous or Injections, Intravenous or Intravenous Administration or Intravenous Administrations or Intravenous Drug Delivery System or Intravenous Drug Delivery Systems or Intravenous Infusion or Intravenous Infusions or Intravenous Injection or Intravenous Injections)).mp. [mp=title, abstract, heading word, drug trade name, original title, device manufacturer, drug manufacturer, device trade name, keyword heading word, floating subheading word, candidate term word] (1344382)
**52**  (Infusion, Parenteral or Infusions, Parenteral or Parenteral Infusion or Parenteral Infusions).mp. (393)
**53**  intravenous.mp. or exp Injections, Intravenous/ or exp Administration, Intravenous/ or exp Infusions, Intravenous/ (1344470)
**54**  exp Infusions, Parenteral/ or parenteral.mp. (926605)
**55**  (Drug Infusion System or Drug Infusion Systems or Infusion System, Drug or Infusion Systems, Drug or Infusion, Drip or Pump, Infusion).mp. (611)
**56**  infusion.mp. (455339)
**57**  51 or 52 or 53 or 54 or 55 or 56 (2082027)
**58**  50 and 57 (14169)
**59**  limit 58 to (human and (article or article in press or conference abstract or conference paper or "conference review" or data paper or editorial or erratum or letter or note or "preprint (unpublished, non-peer reviewed)" or "review" or short survey) and (adolescent or adult or aged )) (8198)

**Supplemental Table 4: Cochrane CENTRAL Search Strategy**

ID Search Hits

#1 opioid$ 31461

#2 MeSH descriptor: [Analgesics, Opioid] explode all trees 10688

#3 #1 or # 2 1349212

#4 opiate$ 6253

#5 MeSH descriptor: [Opiate Alkaloids] explode all trees 13673

#6 #4 or #5 18128

#7 morphine 18295

#8 MeSH descriptor: [Morphine] explode all trees 5959

#9 #7 or #8 18295

#10 oxycodone 3201

#11 MeSH descriptor: [Oxycodone] explode all trees 1207

#12 #10 or #11 3201

#13 fentanyl 19391

#14 MeSH descriptor: [Fentanyl] explode all trees 6726

#15 #13 or #14 20969

#16 remifentanil 6626

#17 MeSH descriptor: [Remifentanil] explode all trees 2157

#18 #16 or #17 6626

#19 alfentanil 1719

#20 MeSH descriptor: [Alfentanil] explode all trees 778

#21 #19 or #20 1719

#22 sufentanil 4680

#23 MeSH descriptor: [Sufentanil] explode all trees 1263

#24 #22 or #23 4680

#25 hydromorphone 1347

#26 MeSH descriptor: [Hydromorphone] explode all trees 497

#27 #25 or #26 1347

#28 #3 or #6 or #9 or #12 or #15 or #18 or #21 or #24 or #27 1360700

#29 MeSH descriptor: [Critical Care] explode all trees 3037

#30 intensive care 53484

#31 (ICU or MICU or SICU or CICU or CCU) 22062

#32 critical care 37638

#33 #29 or #30 or #31 or #32 86878

#34 #28 and #33 59932

#35 ((mechan* or artific*) adj3 (respirat* or ventilat*)) 827

#36 ventilat* or respirat* 146419

#37 CPAP or ncpap or IPPV or APRV 6500

#38 MeSH descriptor: [Ventilators, Mechanical] explode all trees 414

#39 MeSH descriptor: [Respiration, Artificial] explode all trees 9168

#40 #35 or #36 or #37 or #38 or #39 148441

#41 #34 and #40 23765

#42 MeSH descriptor: [Administration, Intravenous] explode all trees 22458

#43 MeSH descriptor: [Infusions, Parenteral] explode all trees 14805

#44 intravenous or parenteral or infusion 162477

#45 infusion 75713

#46 MeSH descriptor: [Infusion Pumps] explode all trees 1648

#47 #42 or #43 or #44 or #45 or #46 162732

#48 #41 and #47 in Cochrane Reviews, Cochrane Protocols, Trials, Editorials, Special Collections 5224

**Supplemental Table 5: WHO ICTRP search strategy**

(opioid* OR morphine OR fentanyl OR oxycodone OR remifentanil OR sufentanil OR alfentanil OR hydromorphone) AND (ventilat* OR (artificial respiration)) AND ((intensive care) OR (critical care))

**Supplemental Table 6: Clinical trials.gov search strategy**

Condition: ventilation OR ventilate OR artificial respiration

Intervention: (opioid* OR morphine OR fentanyl OR oxycodone OR remifentanil OR sufentanil OR alfentanil OR hydromorphone)

**Supplemental Table 7: ‘Web of Science Conference Proceedings Citation Index’ and ‘ProQuest Dissertation and Theses’ search strategy**

Key words: opioid, morphine, fentanyl, oxycodone, remifentanil, sufentanil, alfentanil, hydromorphone, ventilat*, respiration, intensive care, critical care, ICU

**Supplemental Table 8. Definitions of outcomes**

| **Outcomes** | **Definition(s)** |
| --- | --- |
| **Duration of mechanical ventilation** | - Period from the administration of the drug following randomisation, to the actual extubation time. |
| **Changes in ICU pain scores** | - ICU pain scores were measured using a validated tool: Behavioural Pain Scale (BPS), Critical-Care Pain Observation Tool (CPOT), Numeric Pain Rating Scale (NRS) or Visual Analogue Scale (VAS). - Pain was categorised into:   - No pain (BPS=3 or CPOT=0 or NRS=0 or VAS=0-4mm  - Mild pain (BPS=4-6 or CPOT=1-3 or NRS=1-3 or VAS = 5-44mm)  - Moderate pain (BPS=7-9 or CPOT=3-6 or NRS=4-6 or VAS=45-74mm)  - Severe pain (BPS=10-12 or CPOT=6-8 or NRS=7-10 or VAS=75-100mm)   - Changes in pain scores was defined as a change in the categories in the pain scores. |
| **Delirium** | - Delirium incidence was defined as the new cases of delirium at any time over a population during the study. - Delirium prevalence was defined as the number of delirium cases, including the pre-existing cases over a population during a study period. - Delirium occurrence was defined as the presence of delirium at any time during the study, regardless of the presence or absence of delirium prior to study enrolment. Delirium occurrences were reported when there were heterogeneities between studies reporting delirium incidence or prevalence. - Validated delirium screening tools were the confusion assessment method in ICU (CAM-ICU) or the Intensive Care Delirium Screening Checklist. - There was no minimum duration required for delirium. The severity and duration of delirium were reported in this review if they were reported by the source study. |
| **Coma** | - Coma was defined as a Richmond Agitation Scale score (RASS) score ≤ -3 to -5 or a Ramsay sedation score > 4. - The minimum duration required for coma was ≥ 1 ICU day. |
| **Length of stay** | - ICU length of stay was defined as the time from admission to discharge from ICU. - Hospital length of stay was defined as the time from admission to discharge from hospital. |
| **Short term mortality** | - Mortality that occurred in hospital or mortality at longest reported follow up to 28 days after ICU admission. |

Mean is expressed as a mean (± standard deviation). Median is expressed as median (inter-quartile range).

Hr, hour; IV, intravenous; IVP, intravenous push; kg, kilogram; mcg, microgram; min, minute; MV, mechanical ventilation; NR, not reported; PRN, as needed; Q12h, every 12 hours

**Supplemental Table 9: Data extraction pre-piloted form**

Name of study

Authors

Date/Year of publication

Country

Type of study

Date/Year of study and duration of study

Type of ICU

Inclusion and exclusion criteria

Intervention group

- Continuous IV opioids, dose and duration
- Use of non-opioid analgesics or sedatives (drugs, dose and duration)

Control group

- Sedatives (drugs, continuous or non-continuous, dose and duration)
- Non-opioid analgesics (drugs, dose and duration)
- Non-continuous IV opioids (drugs, dose and duration)

Number of participants in each group

Age of participants in each group (mean ± SD)

Proportion of participants in male/female

Baseline severity illness

- Acute Physiology and Chronic Health Evaluation Score (APACHE-II) score
- Sequential Organ Failure Assessment (SOFA) score
- Simplified Acute Physiology Score II (SAPS II) score

Outcomes:

- Duration of mechanical ventilation
  - - Definition
    - Measurement of ventilation duration (mean ± SD or median with interquartile range)
- ICU pain scores reduction
  - - Definition
    - Type of validated tool used (BPS/CPOT/NRS/VAS)
    - Time and interval of measurement
    - Pain scores in each group
    - Differences of pain scores/categories between each group
- Delirium
  - - Definition
    - Type of validated tool used (CAM-ICU or Intensive Care Delirium Screening Checklist)
    - Time and interval of measurement
    - Numbers of patients with or without delirium
    - Duration of delirium (mean ± SD or median with interquartile range)
    - Severity
- Coma
  - - Definition
    - Type of validated tool used (RASS or Ramsay Sedation Scale)
    - Time and interval of measurement
    - Number of patients with or without coma
    - Duration of coma (mean ± SD or median with interquartile range)
- ICU length of stay
  - - Definition
    - Duration of ICU length of stay (mean ± SD or median with interquartile range)
- Hospital length of stay
  - - Definition
    - Duration of hospital length of stay (mean ± SD or median with interquartile range)
- Mortality
  - - Definition
    - Follow-up time after randomisation to identify mortality case
    - Number of mortality cases present in each group

**Supplemental Table 10. Description of opioids and sedative dosing and exposure across intervention and control groups for the trials included in the analysis**

| **Study** | **Intervention** | | **Control** | | **Duration** |
| --- | --- | --- | --- | --- | --- |
|  | **Opioid Infusion Use** | **Sedative Use** | **Sedative Use** | **Opioid/Analgesic Use** |  |
| Anvaripour [55] | Morphine 0.015 mg/kg/hr (start dose) via PCA  Daily dose = NR | Use of sedative NR | Dexmedetomidine 0.7 mcg/kg/hr (start dose) via PCA  Daily dose = NR | Use of ‘as needed’ opioids NR | NR for either arm |
| Wang [46] | Sufentanil 1 mcg/kg/hr (start dose) titrated to pain/sedation goals  Daily dose = NR | Propofol 1 mg/kg/hr (start dose) titrated to sedation goal  Daily dose = NR | Propofol 1 mg/kg/hr (start dose) titrated to sedation goal  Daily dose = NR | Use of ‘as needed’ opioids NR | NR for either arm |
| Liu [54] | Arm 1: Fentanyl 1 mcg/kg/hr (no titration)  Mean daily dose of fentanyl: 96.4 ± 14.1 mg | Arms 1 and 2:  Midazolam 0.02-0.1 mg/kg/hr titrated to sedation goal  Mean daily midazolam dose:  Arm 1= 146.8 ± 31.9 mg  Arm 2= 160.6 ± 33.0 mg | Arms 1 and 2:  Midazolam 0.02-0.1 mg/kg/hr titrated to sedation goal  Mean daily midazolam dose: Control arm = 179.0 ± 43.7 mg | Placebo opioid infusion (both arms) titrated to pain goal  Use of placebo opioid infusion NR | Until extubation |
|  | Arm 2: Remifentanil 1 mcg/kg/hr (no titration)  Mean daily dose of remifentanil = 98.6 ± 25.0 mg |  |  |  |  |
| Lyu [53] | Remifentanil 1 mcg/kg/hr (no titration)  Mean daily dose of remifentanil = 98.6 ± 24.9mg | Midazolam 0.02-0.1 mg/kg/hr titrated to sedation goal  Mean daily midazolam dose = 160.6 ± 33.0 mg | Midazolam 0.02-0.1 mg/kg/hr titrated to sedation goal  Mean daily midazolam dose in control arm = 179.0 ± 43.7mg | Use of opioids or other analgesics NR | Until extubation or up to 7 days after randomization |
| Oliver [52] | Arm 1:  Fentanyl 2.0 mcg/kg/hr (start dose) titrated to pain/sedation goal  Median (IQR) fentanyl rate at sedation interruption prior to extubation = 1.0 (0.9-1.4) mcg/kg/hr | Arm 1:  Midazolam IVP PRN agitation  (dose not specified) | Arms 1 and 2:  Propofol 25-65 mcg/kg/min titrated to sedation goal | Arms 1 and 2:  Morphine IVP for pain when required (dose not specified)  Median (IQR) fentanyl equivalent dose at interruption prior to extubation = 80 (80-160) mcg | NR for either arm |
|  | Arm 2:  Fentanyl 0.5 mcg/kg/hr (start dose) titrated to pain/sedation goals  Median (IQR) fentanyl rate at sedation interruption prior to extubation: 0.5 (0.4-0.7) mcg/kg/h | Arm 2:  Propofol 25-65 mcg/kg/min titrated to sedation goal |  |  |  |
| Maddali [51] | Fentanyl 0.25-1.5 mcg/kg/hr titrated to pain/sedation goal  Median (IQR) total fentanyl dose:  39 (34-45) mcg/kg | Propofol 2-5mg/kg/hr titrated to sedation goal  Median (IQR) total propofol dose = 410 (300-442) mg | Propofol 2-5 mg/kg/hr  Median (IQR) total propofol dose = 285 (237-342) mg | Fentanyl 1-2 μg/kg IVP PRN pain + Diclofenac 75 mg suppository repeated q12h x once PRN  Median (IQR) total fentanyl dose = 16.5 (12-20) mcg/kg | NR for either arm |
| Richman [50] | Fentanyl 0.5 mcg/kg/hr (start dose) titrated to pain/sedation goals | Midazolam 0.03 mg/kg/hr (start dose) titrated to sedation goal | Midazolam 0.03 mg/kg/hr (start dose) titrated to sedation goal | No use of analgesics | Until extubation or up to 72 hrs of data collection |

**Supplemental Table 11. Risk of bias of included trials**

| **First author name and publication year** | **Random sequence generation** | **Allocation concealment** | **Blinding of patients and personnel** | **Blinding outcome assessors** | **Incomplete outcome** | **Selective reporting** | **Other** | **Overall rating** |
| --- | --- | --- | --- | --- | --- | --- | --- | --- |
| Anvaripour, A et al., 2018 | Low | Probably low | Probably high | Probably high | Low | Probably low | Probably low | High |
| Liu D et al., 2017 | Low | Probably low | Low | Probably high | Low | Probably low | Probably low | High |
| Lyu, J et al., 2015 | Low | Probably low | Probably high | Probably high | Unclear | Probably low | Probably low | High |
| Maddali M, et al., 2006 | Low | Probably low | Probably high | Probably high | Low | Probably low | Probably low | High |
| Oliver W et al., 2011 | Low | Probably low | Low | Low | Low | Probably low | Probably low | Low |
| Richman PS et al., 2006 | Probably low | Probably low | Probably high | High | Low | Probably low | Probably low | High |
| Wang J et. al., 2021 | Probably low | Probably low | Probably high | Probably high | Low | Probably low | Probably low | High |

**Supplemental Table 12:** **GRADE Summary of findings table**

| **Certainty assessment** | | | | | | | **No of patients** | | **Effects** | | **Certainty** |
| --- | --- | --- | --- | --- | --- | --- | --- | --- | --- | --- | --- |
| **No of studies** | **Study design** | **Risk of bias** | **Inconsistency** | **Indirectness** | **Imprecision** | **Other considerations** | **Interventions** | **Comparisons** | **Relative (95% CI)** | **Absolute (95% CI)** |  |
| Duration of mechanical ventilation | | | | | | | | | | | |
| 3 | randomised trials | serious^a^ | not serious | serious^b^ | very serious^c^ | none | 157 | 164 | - | **MD 3.63 hours more** (2.27 more to 4.99 more) | ⨁◯◯◯ Very low^a,b,c^ |
| Reduction in pain score | | | | | | | | | | | |
| 5 | randomised trials | serious^a^ | serious^d^ | not serious | not serious | none | Total of 547 patients. (Intervention: 335 patients Control: 212 patients)  Out of five studies, four studies reported no statistically significant reduction in pain score between intervention and comparison group and one study reported statistically significant reduction in pain score in intervention group at 24 hours of treatment and after extubation. | | | | ⨁⨁◯◯ Low^a,d^ |
| Delirium | | | | | | | | | | | |
| 3 | randomised trials | serious^a^ | not serious | serious^e^ | serious^f^ | none | 39/175 (22.3%) | 62/140 (44.3%) | **OR 0.28**  (0.16 to 0.47) | **261 fewer per 1000**  (from 330 fewer to 171 fewer) | ⨁◯◯◯ Very low^a,e,f^ |
| Prevalence of coma | | | | | | | | | | | |
| 1 | randomised trial | serious^a^ | not serious | serious^g^ | serious^f^ | none | 1/17 (5.9%) | 3/13 (23.1%) | **OR 0.20**  (0.02 to 2.29) | **174 fewer per 1000**  (from 225 fewer to 176 more) | ⨁◯◯◯ Very low^a,f,g^ |
| ICU length of stay | | | | | | | | | | | |
| 3 | randomised trials | serious^a^ | not serious | serious^b^ | very serious^h^ | none | 212 | 146 | - | **MD 0 day**  (0.03 lower to 0.04 higher) | ⨁◯◯◯ Very low^a,b,h^ |
| Hospital length of stay | | | | | | | | | | | |
| 1 | randomised trial | not serious | not serious | not serious | serious^f^ | none | 72 | 41 | - | **MD**  **0 days**  (0 to 0) | ⨁⨁⨁◯ Moderate^f^ |
| Short-term mortality | | | | | | | | | | | |
| 3 | randomised trials | serious^a^ | not serious | serious^e^ | serious^f^ | none | 16/175 (9.1%) | 27/140 (19.3%) | **OR 0.41** (0.21 to 0.80) | **104 fewer per 1000** (from 145 fewer to 32 fewer) | ⨁◯◯◯ Very low^a,e,f^ |

**CI:** confidence interval; **MD:** mean difference; **OR:** odds ratio

**Explanations**

a. Unclear and difficulty in blinding of outcome assessor due to clinical environment

b. Two out of three studies used midazolam infusion alone as comparator group. Midazolam is not the first line sedative agent used for mechanically ventilated ICU adult patients.

c. Two out of three studies have wide 95% confidence interval (CI), with the upper limit and lower limit of CI suggest both benefit and harm.

d. Varied in the participants included in studies. Three studies included only elective cardiac surgery patient, one study included emergency and elective surgery patients, and one study included medical patients. Four studies used VAS to measure pain, whilst one study used CPOT to measure pain.

e. Two out of three studies used midazolam infusion alone as comparator group, whilst one study used dexmedetomidine and propofol as comparator. Midazolam is not the first line sedative agent used for mechanically ventilated ICU adult patients.

f. Small sample size.

g. The study used midazolam infusion alone as comparator group. Midazolam is not the first line sedative agent used for mechanically ventilated ICU adult patients.

h. Small sample size. The upper limit and lower of 95% CI of all three studies suggest both benefit and harm

**Supplemental Table 13. Systematic review of five trials examining continuous IV opioids (versus non-continuous IV opioids) and pain score reduction**

| **First author name and publication year** | **Pain Score Scale Used** | **Treatment arms** | **Pain score (corresponding severity)** | | | | | **Significance** |
| --- | --- | --- | --- | --- | --- | --- | --- | --- |
|  |  |  | **Before treatment** | **Within 1 hour of treatment** | **12 hours of treatment** | **24 hours of treatment** | **Post- extubation** |  |
| Anvaripour, A et al., 2018 | VAS | Intervention: Morphine | NR | VAS mean = 4.42  [mild pain] | VAS mean = 3.78  [mild pain] | VAS mean = 3.18  [mild pain] | - | No significant difference between groups. |
|  |  | Control: Dexmedetomidine | NR | VAS mean =  5 [moderate pain] | VAS mean= 4.15  [mild pain] | VAS mean = 3.58  [mild pain] | - |  |
| Liu, D et al., 2017 | CPOT | Intervention Arm 1: fentanyl + midazolam | CPOT median = 4 (3,4)  [moderate pain] | After sedation CPOT median score = 0 (0,0) [no pain] | | | | No significant difference between groups. |
|  |  | Intervention Arm 2: remifentanil + midazolam | CPOT median = 3 (2,4)  [moderate pain] | After sedation CPOT median score = 0 (0,0) [no pain] | | | |  |
|  |  | Control: midazolam + saline | CPOT median = 4 (3,4)  [moderate pain] | After sedation CPOT median score = 0 (0,0) [no pain] | | | |  |
| Maddali, M, et. al, 2006 | VAS | Intervention Arm 1: Fentanyl + Propofol |  | Post extubation 45 mins VAS median = 5 mm (0-70) [mild pain]  Post extubation 6 hours VAS median = 4mm (0-70) [no pain]  Post extubation 12 hours VAS median = 4mm (0-50) [no pain] | | | | No significant difference between groups. |
|  |  | Intervention Arm 2: Remifentanil + Propofol |  | Post extubation 45 mins VAS median = 4 mm (0-40) [no pain]  Post extubation 6 hours VAS median = 4mm (0-50) [no pain]  Post extubation 12 hours VAS median = 4mm (0-30) [no pain] | | | |  |
|  |  | Control: Propofol + Diclofenac 75mg |  | Post extubation 45 mins VAS median = 4 mm (0-30) [no pain]  Post extubation 6 hours VAS median = 4mm (0-50) [no pain]  Post extubation 12 hours VAS median = 4mm (0-20) [no pain] | | | |  |
| Oliver, W et al., 2011 | VAS | Intervention Arm 1: Fentanyl + Midazolam |  | Study reported no difference in percentage of patients with VAS ≥ 4 between groups (actual data not reported). | | | | |
|  |  | Intervention Arm 2: Propofol + Fentanyl prn |  |  |  |  |  |  |
|  |  | Control: Propofol + Morphine PRN |  |  |  |  |  |  |
| Wang, J et al, 2021 | VAS | Intervention: Sufentanil + Dexmedetomidine | VAS mean = 4.77±1.35 [moderate pain] |  |  | VAS mean = 3.86±0.94  [mild pain] | VAS mean =2.60±0.81  [mild pain] | Significant reduction in pain with opioid group but no clinically significant reduction in pain severity. |
|  |  | Control: Dexmedetomidine | VAS mean= 4.91±1.15 [moderate pain] |  |  | VAS mean = 4.31±0.83  [mild pain] | VAS mean= 3.14±0.73  [mild pain] |  |

| **First author name and publication year** | **Pain Score Scale Used** | **Treatment arms** | **Pain score (corresponding severity)** | | | | | **Significance** |
| --- | --- | --- | --- | --- | --- | --- | --- | --- |
|  |  |  | **Before treatment** | **Within 1 hour of treatment** | **12 hours of treatment** | **24 hours of treatment** | **Post- extubation** |  |
| Wang, J et al., 2021 | VAS | Intervention: Sufentanil + Dexmedetomidine | VAS mean = 4.77±1.35 [moderate pain] |  |  | VAS mean = 3.86±0.94  [mild pain] | VAS mean =2.60±0.81  [mild pain] | Significant reduction in pain with opioid group but no clinically significant reduction in pain severity. |
|  |  | Control: Dexmedetomidine | VAS mean= 4.91±1.15 [moderate pain] |  |  | VAS mean = 4.31±0.83  [mild pain] | VAS mean= 3.14±0.73  [mild pain] |  |

**Supplemental Figure 1.** **Forest plot examining the relationship between continuous IV opioid (versus no continuous IV opioid) and mean VAS score at 24 hours of treatment using fixed effect inverse-variance methods.**

**
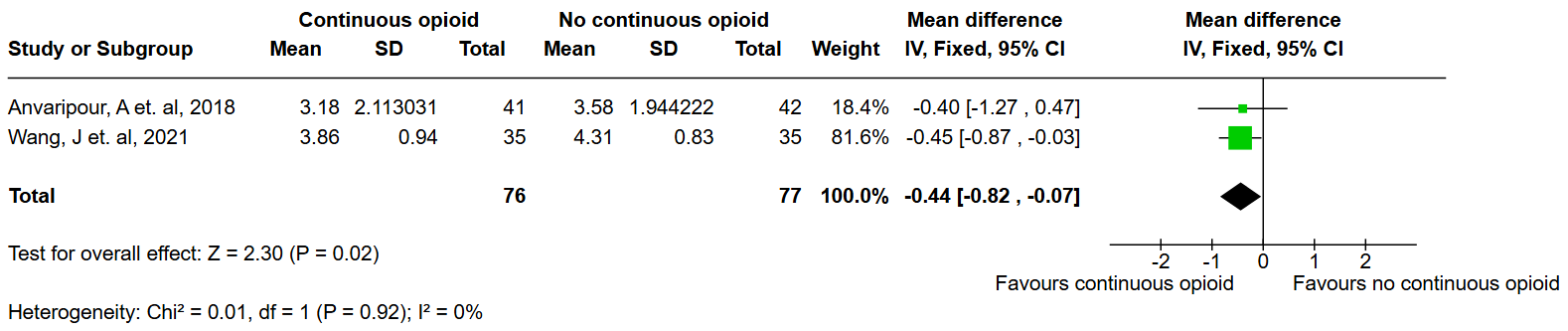
**

**Supplemental Figure 2. Forest plot examining the relationship between continuous IV opioid (versus no continuous IV opioids) and hours with delirium using fixed-effect inverse variance methods.**


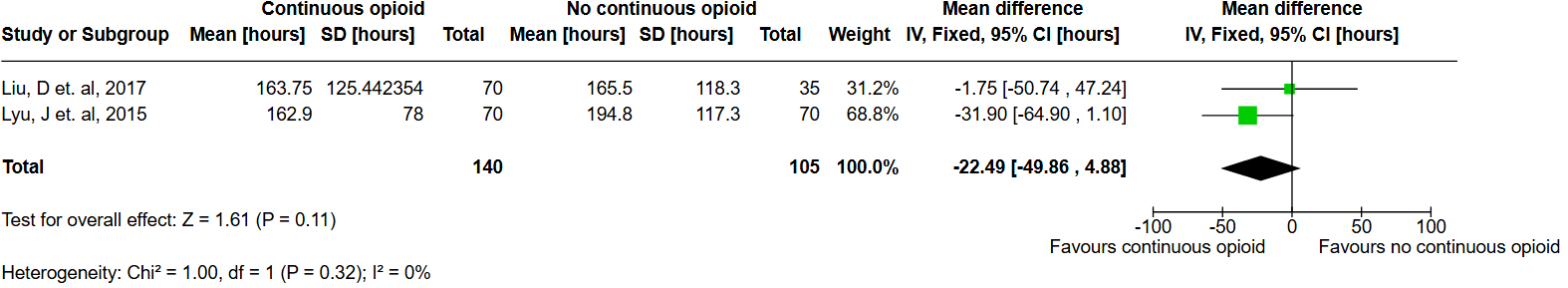


**Supplemental Figure 3. Forest plot examining the relationship between continuous IV opioid (versus no continuous IV opioid) and ICU length of stay using REML random-effect Wald-type method.**

**
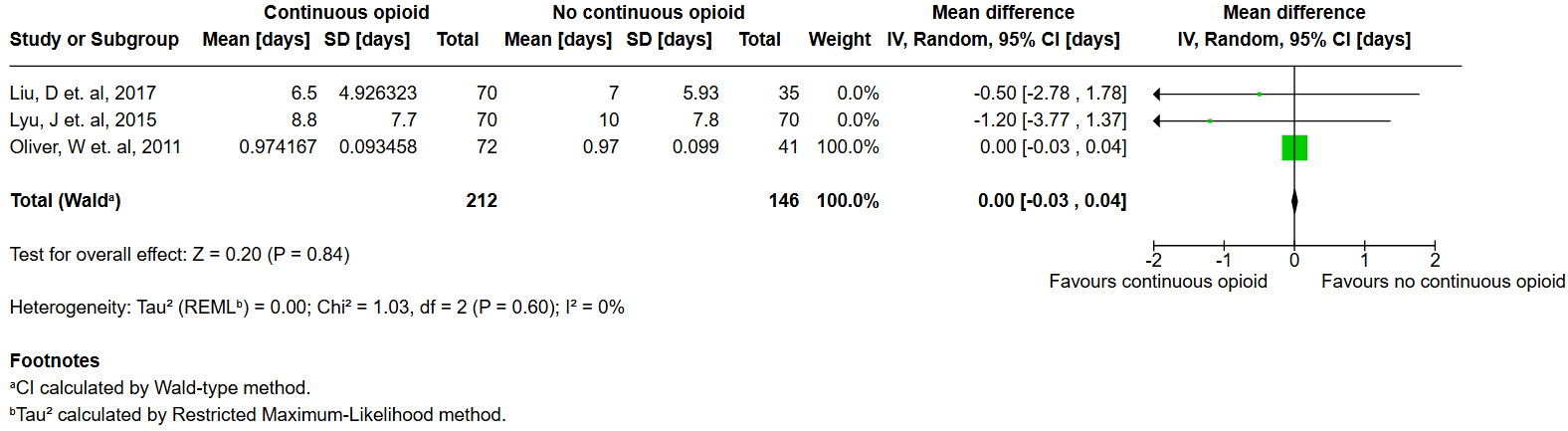
**

**Supplemental Figure 4. Forest plot examining the relationship between continuous IV opioid (versus no continuous IV opioid) and duration of mechanical ventilation using Hartung-Knapp-Sidik-Jonkman method.**


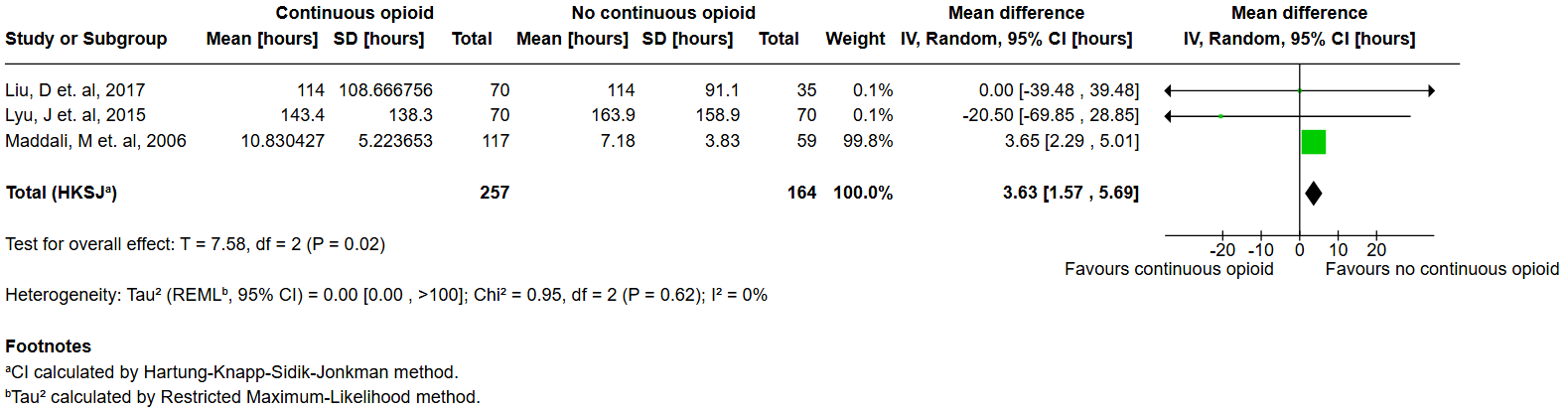


**Supplemental Figure 5. Forest plot examining the relationship between continuous IV opioid (versus no continuous IV opioid) and delirium occurrence using Peto method.**


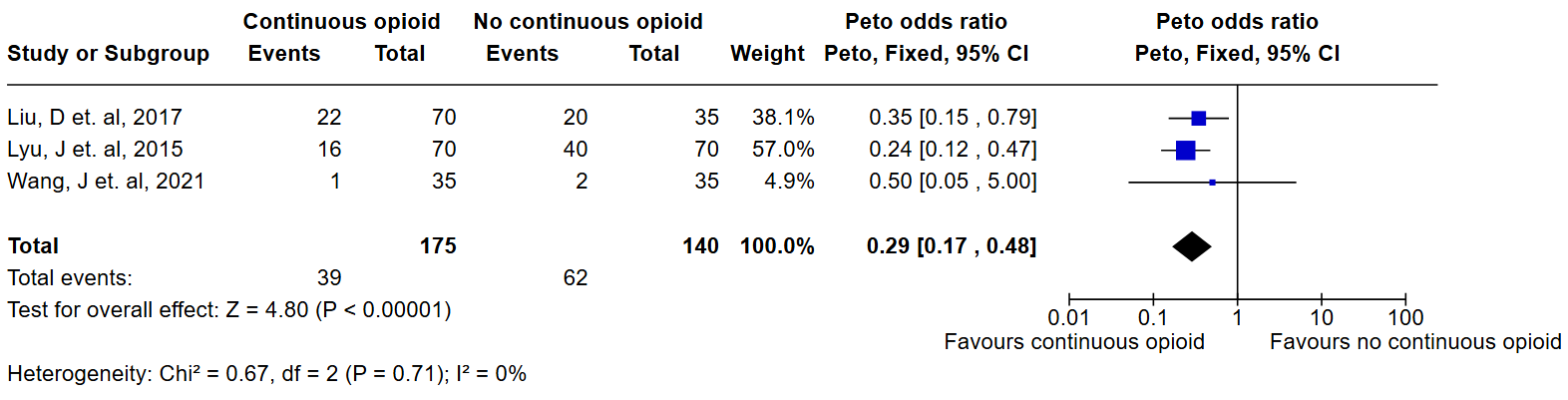


**Supplemental Figure 6. Forest plot examining the relationship between continuous IV opioid (versus no continuous IV opioid) and short-term mortality using Peto method.**


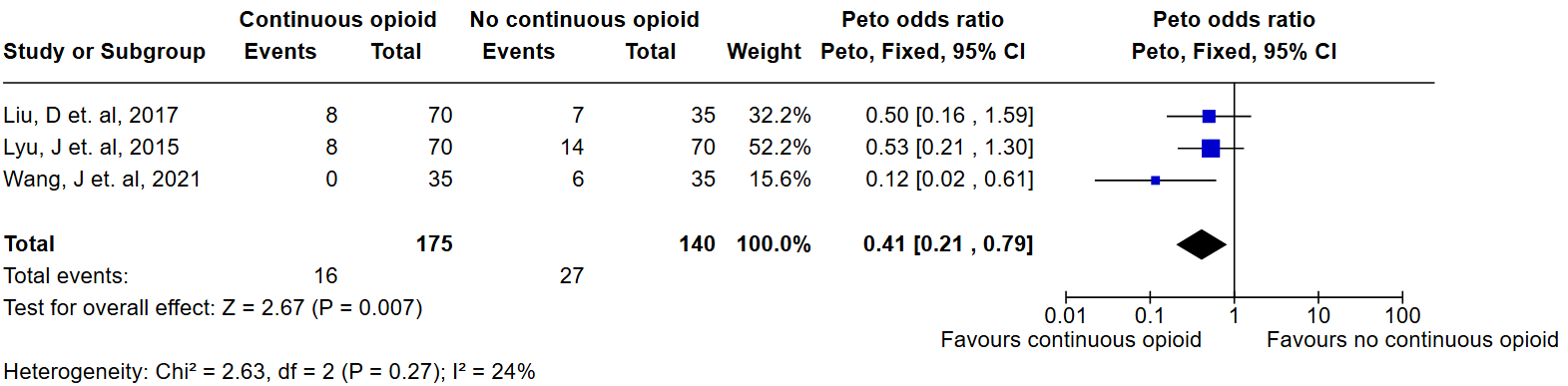


**Supplemental Table 14. Results from sensitivity analyses evaluating patients undergoing cardiac surgery, receiving propofol or dexmedetomidine, longer opioid infusion durations, or greater daily opioids for each study outcome**

| **Outcomes** | **Studies including post-cardiac surgery patient only** | **Studies using propofol and dexmedetomidine as comparator only** | **Studies using opioid for ≥48 hours only** | **Studies using average daily dose of opioid of ≥200mg MME only** |
| --- | --- | --- | --- | --- |
| **Duration of mechanical ventilation** | | | | |
| **Mean difference (MD)**  **(95% CI)**  **p-value** |  |  | -8.0  (-38.8, 22.8)  p >0.05 | -8.0  (-38.8, 22.8)  p >0.05 |
| **Number of studies** |  |  | 2 | 2 |
| **Number of participants** |  |  | 245 | 245 |
| **Reduction in pain scores** | | | | |
| **Overall effect** | No statistically significant difference between two arms | No statistically significant difference between two arms apart from one study |  |  |
| **Number of studies** | 3 | 4 |  |  |
| **Number of participants** | 372 | 442 |  |  |
| **Delirium occurrence** | | | | |
| **Odds ratio (95% CI)**  **p-value** |  |  |  |  |
| **Number of studies** |  |  |  |  |
| **Number of participants** |  |  |  |  |
| **ICU length of stay** | | | | |
| **Mean difference (MD)**  **(95% CI)**  **p-value** |  |  | -0.81  (-2.51, 0.90)  p=0.35 | -0.81  (-2.51, 0.90)  p=0.35 |
| **Number of studies** |  |  | 2 | 2 |
| **Number of participants** |  |  | 245 | 245 |
| **Short term mortality** | | | | |
| **Odds ratio (95% CI)**  **p-value** |  |  |  |  |
| **Number of studies** |  |  |  |  |
| **Number of participants** |  |  |  |  |

**Supplemental Table 15. Results from sensitivity analyses excluding potentially overlapping studies for each study outcome**

| **Outcomes** | **Analyses excluding Liu [54]** | **Analyses excluding Lyu [53]** |
| --- | --- | --- |
| **Duration of mechanical ventilation** | | |
| **Mean difference (MD)**  **(95% CI)**  **p-value** | 3.63 (2.27, 4.99)  p <0.01 | 3.65 (2.29, 5.01)  p <0.01 |
| **Number of studies** | 2 | 2 |
| **Number of participants** | 316 | 281 |
|  |  |  |
| **Reduction in pain scores** | | |
| Not applicable as Lyu, J et al [27] did not report on pain scores. | | |
|  |  |  |
| **Incidence of delirium** | | |
| **Odds ratio (95% CI)**  **p-value** | 0.24 (0.12, 0.48)  p <0.01 | 0.36 (0.16, 0.79)  p =0.01 |
| **Number of studies** | 2 | 2 |
| **Number of participants** | 210 | 175 |
|  |  |  |
| **ICU length of stay** | | |
| **Mean difference (MD)**  **(95% CI)**  **p-value** | 0.00  (-0.03, 0.04)  p =0.84 | 0.00  (-0.03, 0.04)  p =0.83 |
| **Number of studies** | 2 | 2 |
| **Number of participants** | 253 | 218 |
|  |  |  |
| **Short term mortality** | | |
| **Odds ratio (95% CI)**  **p-value** | 0.36  (0.15, 0.85)  p =0.02 | 0.32  (0.12, 0.84)  p =0.02 |
| **Number of studies** | 2 | 2 |
| **Number of participants** | 210 | 175 |
| **Comments** | I^2^ equals to 47% (moderate heterogeneity) when either of the trials were excluded.  When using inverse-variance random-effect Wald-type model, there were no statistically significant difference in short-term mortality between both groups when either Liu, D et. al (OR=0.29, 95% CI= 0.05,1.81) or Lyu, J et. al (OR=0.29, 95% CI= 0.05,1.81) was excluded. | |
